# Ethics Reporting in Biospecimen and Genetic Research: Current Practice and Suggestions for Changes

**DOI:** 10.1371/journal.pbio.1002521

**Published:** 2016-08-02

**Authors:** William Wei Lim Chin, Susanne Wieschowski, Jana Prokein, Thomas Illig, Daniel Strech

**Affiliations:** 1 Institute for History, Ethics, and Philosophy of Medicine, Hannover Medical School, Hannover, Germany; 2 Hannover Unified Biobank, Hannover Medical School, Hannover, Germany

## Abstract

Modern approaches for research with human biospecimens employ a variety of substantially different types of ethics approval and informed consent. In most cases, standard ethics reporting such as “consent and approval was obtained” is no longer meaningful. A structured analysis of 120 biospecimen studies recently published in top journals revealed that more than 85% reported on consent and approval, but in more than 90% of cases, this reporting was insufficient and thus potentially misleading. Editorial policies, reporting guidelines, and material transfer agreements should include recommendations for meaningful ethics reporting in biospecimen research. Meaningful ethics reporting is possible without higher word counts and could support public trust as well as networked research.

## Abstract 2

This Perspective shows that while ethics reporting on consent and approval for biospecimen research is widespread, for the majority of cases it is not meaningful; the authors make pragmatic suggestions of substantial changes needed to remedy this.

## Introduction

High-quality biobanks are important resources for health research, including basic and genetic research, investigations in personalized or stratified medicine (genetic and other biomarkers), and research in widespread diseases. The development of large-scale, population-based as well as disease-specific biobanks has come with new ethical challenges. New consent procedures are needed because big as well as small research biobanks are increasingly practicing long-term storage of biospecimens and data [1,2]. These stored materials and data could be used for future research projects that are unspecified—and even to some extent unforeseen—at the time of the donor’s consent. Some biomaterial donors, therefore, are asked to give consent to a framework for future research of certain types (“broad consent”) instead of the standard consent to a single research project (“project-specific consent”) [3,4]. Other studies might employ so-called “dynamic consent” models [5]. Single research projects based on project-specific consent still exist in parallel.

The increased use of the broad consent model also affects how competent bodies review and approve biospecimen research. To grasp these implications, it is helpful to distinguish two steps in the review and approval of biospecimen research operating under a broad consent model [6]. First, the biobanking itself can be approved (“bank approval”). This approval concerns the “framework of oversight procedures,” such as the broad consent form, data protection model, and other policies relevant to the biobank’s governance. Second, the same or another competent body can approve specific projects that require the release of particular biomaterials and data (“release approval”). The other common scenario is a single research project that collects, analyzes, and then disposes of all remaining specimens. Such single research projects still work appropriately with a standard ethics approval.

Meaningful ethics reporting in this area needs to go beyond standard sentences such as “consent was obtained from all participants” and “the project was approved by the local ethics committee.” From an ethical viewpoint, it is unclear whether “approval” comprised both the banking and the use of material or only one. Thus, the reporting becomes meaningless or even misleading in cases in which, for example, the initial approval given did not include the reuse of biospecimens.

The “Biospecimen Reporting for Improved Study Quality” (BRISQ) guideline [7], the “Guideline to Standardize the Citation Of BioResources in journal Articles” (COBRA) [8], and the “Minimum Information About BIobank data Sharing” (MIABIS) guideline [9] do not include specific requirements for ethics reporting. Likewise, neither the International Committee of Medical Journal Editors (ICMJE) nor the Committee on Publication Ethics (COPE) currently provide specific guidance on ethics reporting in biospecimen research.

Cheah et al. found that 35%–40% of studies with human biospecimens published in two cancer journals between 1998 and 2010 reported on informed consent and that reporting frequency of ethics approval increased from 25% to 60% [10]. However, this study did not assess or discuss whether ethics reporting was meaningful in light of the above-described complexity of consent and approval.

## Status Quo: Ethics Reporting in Biospecimen Research

We systematically screened ethics statements from a total of 120 articles reporting on analyses of human biospecimens published between 2014 and 2015 in *PLOS Genetics* (*n* = 30), *The New England Journal of Medicine* (*n* = 30), *Nature Genetics* (*n* = 30), and *Nature Medicine* (*n* = 30). S1 Table presents the extracted ethics reporting of all 120 papers. S1 Text presents further details on the search and selection of studies and on the analysis of ethics statements.

The proportion of articles derived from biobank studies was 47% (*n* = 56), from clinical drug trials 16% (*n* = 19), and from other clinical studies 38% (*n* = 45). Only 3 studies (3%) lacked any reporting of either consent or approval. 20% (*n* = 24) reported on only one category.

A total of 87% (104/120) of articles reported at least some information on consent. Of these 104 articles, 41% (43/104) reported additional details such as proxy consent, timing of consent, consent obtained from another study or project, or data protection specifications. Only 8% (*n* = 8) of the 104 consent-reporting articles provided information that helped to understand whether the consent was broad or project-specific (S1 Table).

Ninety-three percent (112/120) of the articles reported at least some information on approval. Of these 112 articles, 79% (89/112) reported additional details, such as the name of the institutional review board (IRB) or the approval protocol number. Only 9% (*n* = 10) of the 112 articles provided information that helped to understand whether the approval was granted for the banking of specimens for future research, for a specific project, or for both. Of these 10 articles, 2 reported on bank approval, 4 articles reported on study-specific approvals, and 4 articles reported on both bank and study approvals (S1 Table).

In brief, most papers on biospecimen research reported on consent and approval but did it in a nonmeaningful way. We must stress that nonmeaningful ethics reporting does not imply inadequate consent and approval processes. For a recent status quo analysis of consent procedures in biobank research, see [11].

Our study included four articles that were all based on the same biobank, deCODE Genetics. The reporting on consent and approval differed substantially across the four articles. Two articles reported details that helped the reader to understand the broad consent, biobank approval, and project-specific approval involved. The other two studies only used standard phrases on consent and approval that were not helpful for an appropriate understanding of the consent and approval issues.

It is interesting to see that some authors already realized the need to report more specifically on consent and approval, even though there is as yet no official guidance. Some examples and variations of meaningful reporting on consent and approval are presented and commented on in Table 1. In the majority of cases, unfortunately, even the more detailed information did not allow us to determine the type of consent or approval. We illustrate this with typical examples in S2 Table.

**Table 1:** Selected ethics statement (see also S1 Table) with specifying reporting on informed consent or ethics approval or both.

Example of original ethics statement	Explanation		Example of modified, clearer ethics statement*
	Strength	Weakness	
All DNA samples were collected after approval from relevant institutional research ethics committees. Review boards of all contributing institutions approved all protocols and informed consent for sharing of data and sample collection; appropriate informed consent was obtained from all subjects and families. (NG14, S1 Table)	The phrase “approved all protocols and informed consent for sharing of data and sample collection” together with “appropriate informed consent was obtained” indicates that the consent was broad and that the approval included both bank approval (“sharing of data and sample collection”) and study-specific approval (“all protocols”).	It is unclear whether remaining biospecimens were disposed of at the end of the study or stored for future research. It is also unclear what “subjects and families” means. Does “family” imply “legal proxy” and is “and” accurate, or should it be “subjects or their proxies”?	This study used DNA samples collected at all contributing institutions. The sample collection and sharing of data as well as the specific study protocols were approved by the local review boards from all institutions in [year]. All subjects or their legal proxies gave broad consent to future research with their samples and data, without restriction.
Ethical approval for the GLACIER Study was obtained from the Regional Ethical Review Board in Umeå, Sweden. The Ethics Committee at Lund University approved the MDC study. All participants provided written informed consent as part of the VHU. (note: VHU = Väserbotten Health Survey, prospective population based cohort study). (PG25, S1 Table)	The information “VHU = prospective cohort study” together with the information that study approvals were obtained for GLACIER and MDC indicates that the consent must have been broad and the approvals were study-specific.	It remains unclear whether there was a “bank approval” for the VHU.	The GLACIER and MDC studies used samples from the VHU. The VHU was approved by the ethics committee at [xxx] in [year]. All VHU participants gave broad consent to future research with their samples and data, without restrictions. The GLACIER study was approved by [xxx] in [year]; the MDC study was approved by [xxx] in [year]. Remaining samples in the GLACIER and MDC studies were disposed of.
This study is based on whole-genome sequence data from the white blood cells of 2,636 Icelanders participating in various disease projects at deCODE Genetics (Supplementary Tables 1 and 2)…All participating individuals, or their guardians, gave their informed consent before blood samples were drawn…This integration is performed without the genotypes being stored. All sample identifiers were encrypted in accordance with the regulations of the Icelandic Data Protection Authority. Approval for these studies was provided by the National Bioethics Committee and the Icelandic Data Protection Authority. (NG5, S1 Table)	The phrase “participating in various disease projects at deCODE” indicates that the consent must have been broad.	Readers unfamiliar with the deCODE study cannot infer which study was approved: the deCODE study, the present study, or both.	This study used data from deCODE Genetics. deCODE Genetics was approved by [xxx] in [year]. All deCODE participants gave broad consent to future research with their samples and data, without restrictions. This study was approved by [xxx] in [year].
We conducted this study using data and DNA samples from 16 case–control studies and cohort studies. All study participants provided written informed consent for genetic studies. The institutional review boards at the Broad Institute and each participating site approved the study protocols. (NE9, S1 Table)	The phrase “consent for genetic studies” indicates that the consent was most probably broad and allowed future genetic studies. Together with the information that the samples stem from other studies, the approval information indicates that both the banking of samples in former studies was approved (bank approval) and the reuse in the present study was approved (study-specific approval).	It remains unclear whether the consent to genetic research in the 16 earlier studies explicitly included future research and, similarly, whether the approval of the 16 earlier studies approved the banking for future reuse. Even if there was no broad consent or banking approval, the present study could be approved for good reasons, but these reasons should then be mentioned explicitly.	This study used samples and data from 16 case–control studies and cohort studies. All case–control studies and cohort studies were approved by local ethics committees in [years]. All participants from all 16 case–control studies and cohort studies gave broad consent to future research with their samples and data without restrictions. (Or, alternatively: Participants from all 16 case–control studies and cohort studies consented to genetic studies in the original studies, but did not provide explicit broad consent on future genetic research without restrictions). This study was approved by the IRB at the Broad Institute in [year]. This study was approved despite lack of broad consent because…
The study was prospectively approved by the NHS North West Research Ethical Committee. Clinical and demographic data were collected. During this period, we initiated our CDX study, and 11 patients provided additional informed consent that specified their samples could be used for in vivo studies and genetic analysis in accordance with UK regulatory requirements. (NM27, S1 Table)	The phrase “informed consent that specified their samples could be used for in vivo studies and genetic analysis” together with the information that this consent only covers 11 patients selected for the CDX study indicates that the consent was study-specific.	It remains unclear whether the mentioned approval included the “additional” CDX study and whether the samples of the 11 patients were stored for potential reuse in future projects. If so, then the consent should be classified as broad.	This study used samples from 11 patients who gave study-specific consent to the use of their samples for in vivo studies and genetic analysis. This study was explicitly approved within the approval of the [xxx] study by the ethics committee at [xxx] in [year]. Remaining samples were disposed of.
Saliva samples were collected in Yemen in 2007 with informed consent under Western IRB approval, Olympia, WA. Subsequent analysis of anonymized SNP data was approved by the Lehman College IRB. (NG26, S1 Table)	This text clearly indicate two separate approvals by two independent IRBs—one approval for the sample collection (bank approval) and another for the use in the present study (study-specific approval).	It remains unclear where the samples were stored and whether the consent was broad enough to allow the reuse.	This study used samples from the biobank [xxx]. The biobank [xxx] was approved by the IRB (Olympia, WA) in [year]. All sample donors gave broad consent to future research on their samples and data, without restrictions. This study was approved by the Lehman College IRB in [year]. Remaining samples were disposed of.

* Our example statements are examples of clearer ethics reporting, but we did not check with the authors whether our examples statements correctly characterize the respective studies.

Besides the need to report more specifically on the type of consent and approval, our analysis also discovered that some researchers further report on additional ethical issues such as transborder use, data protection, and handling of protected health information. See Table 2 for examples of such additional ethics statements.

**Table 2:** Examples of additional details on consent and approval extracted from ethics statements.

Type of details on ethics reporting	Explanation	Example
Approval of data protection model	A core ethical issue in biospecimen research is data protection. One might argue that the ethics approval for biobanks or single projects that collect, store, and use biospecimens should explicitly include a review of the data protection model used.	Several studies explicitly mentioned this issue, for example: “After the approvals from ethics committees and data-protection agencies were obtained…”(NE18, S1 Table)
Approval of and/or consent to transborder use	Another core ethical issue in biospecimen research is data protection and material transfer. Data protection differs internationally. A sample donor consenting to storage and use of samples will assume that this happens under national standards for data protection. If the sample is used in other countries with lower data protection standards, the donor should be informed about this and the approval should explicitly include this element.	One study explicitly mentioned that the approval included “…to ship samples out of Brazil…” (PG16, S1 Table)
Approval of and/or consent to handling of protected health information	Data protection is needed to minimize privacy breaches. Such privacy breaches are most important for so-called “protected health information” (that is, health information that might be of relevance to employers or health insurers). One might argue that ethics approval should explicitly include a review of the handling of such protected health information.	One study explicitly mentioned that “…protected health information was reviewed according to Health Insurance Portability and Accountability Act (HIPAA) guidelines”. (NM24, S1 Table)

We also found differences in the justifications of why no informed consent was needed for anonymized or de-identified samples. One paper, for example, just mentioned that samples were de-identified and that their study was approved. Others explicitly justified research with anonymized samples via project-specific conditions and safeguards. One study, for example, highlighted that samples “were not and will not be used for any other purpose.” Some mentioned reasons for waivers of consent that would need more explanation. For example, it remains unclear why no consent is needed for research with “discarded clinical material.” The fact that personalized material was discarded or “left-over” after surgery does not directly justify research without consent. Similarly unclear cases for research without consent were research with “cadaveric organ transplant donors.” Recent debates and interview research with IRB leaders in the United States highlighted the uncertainties on whether anonymization in biobank research still suffices for waivers of consent [12].

## A Roadmap for Improving Meaningful Ethics Reporting

How can the described challenges be addressed in an ethical and pragmatic manner? First, we recommend that ICMJE and COPE as well as individual journals revise their policies on ethics reporting and ask for more meaningful information on:

1. whether project-specific consent, broad consent, or any other consent type was obtained from biospecimen donors; and
2. whether the biobank that collected, stored, and/or released the biospecimen was approved, by which body it was approved (and if not, why not), and whether the particular project was approved and by which body (and if not, why not). If no separate biobank was involved (that is, the research project collected, stored, used, and then disposed of all remaining biospecimens), then this should be explicitly mentioned to avoid misunderstandings.

Furthermore, it is helpful in most cases to know whether the samples used in the study were disposed of at the end of the study or whether remaining samples were stored for reuse. In Table 1, we demonstrated how more meaningful ethics reporting might look without necessarily increasing the word count. Our examples of clearer ethics reporting in Table 1 were restricted to basic information. Future discussion and meta-research should clarify whether even more reporting is needed in particular cases (for example, on whether consent on transborder use of samples was obtained or whether the study’s data protection model was explicitly approved).

Reporting guidelines on biospecimen research (e.g., BRISQ, COBRA, MIABIS) should be revised to add these requirements. We also recommend that each individual biobank include an ethics reporting template into their material transfer agreements to avoid variation in reporting by studies linked to the same biobank.

We recommend a consensus statement by researchers, journal editors, ethicists, and other relevant stakeholders in this regard. More specific reporting on consent and approval in scientific publications should not only be considered as an intrinsic component of good professional conduct in biobank research but could also support the harmonization of biobank research and will inform the reader about opportunities for cooperation.

## Supporting Information

S1 Table Ethics reporting for informed consent and ethics approval by 120 articles extracted from *PLOS Genetics*, *The New England Journal of Medicine*, *Nature Genetics*, and *Nature Medicine*. (DOCX)

S2 Table Selected ethics statements with nonmeaningful reporting on consent, approval, or both. (DOCX)

S1 Text Detailed descriptions of the methods and limitations. (DOCX)

## Abbreviations

BRISQ: Biospecimen Reporting for Improved Study Quality
COBRA: Citation Of BioResources in journal Articles
COPE: Committee on Publication Ethics
ICMJE: International Committee of Medical Journal Editors
IRB: institutional review board
MIABIS: Minimum Information About BIobank data Sharing
