# Supplementary material for: Ethics Reporting in Biospecimen and Genetic Research: Current Practice and Suggestions for Changes
Source: PLoS Biol. 2016 Aug 2;14(8):e1002521. doi: 10.1371/journal.pbio.1002521 (PMC4970810; doi:10.1371/journal.pbio.1002521)
Supplement: S1 Table — (DOCX) [file pbio.1002521.s001.docx]

| **No.** | **Article** | **Ethics statement** | **Informed Consent** | | | | | | | | | **Ethics approval** | | | | | | | | |
| --- | --- | --- | --- | --- | --- | --- | --- | --- | --- | --- | --- | --- | --- | --- | --- | --- | --- | --- | --- | --- |
|  |  |  | **Reporting of whether  informed consent was obtained** | | **Reporting of additional details regarding the informed consent**  *(i.e. proxy consent, timing of consent, consent part of another study/project, specific use of biospecimen, data protection specifications)* | | **Reporting of details whether the consent was broad or/and specific**  *(i.e. whether consent was given to participate only to the specific project or/and consent was given to bank specimens for research)* | | **Type of  informed consent** | | | **Reporting of ethics approval** | | **Reporting of additional details regarding the ethics approval**  *(i.e. name of IRB, protocol number)* | | **Details of ethics approval**  *(i.e. whether approval was given only to the specific project or/and approval that specimens were banked for research)* | | **Type of ethics approval** | | |
|  |  |  | Reported | Not reported | Reported | Not reported | Reported | Not reported | Broad | Specific | Both (Broad + Specific) | Reported | Not reported | Reported | Not reported | Reported | Not reported | Bank approval (Broad) | Study approval (Specific) | Both (Broad + Specific) |
| 1 | PG1 | The human study was approved by the Children’s Hospital of Wisconsin Institutional Review Board (protocol numberCHW03/56) with written informed consent obtained from each participant and/or their legal representative, as appropriate. This study also utilized data generated by the UK10K Consortium, derived fromsamples from UK10K_Rare_Coloboma (EGA Study ID: EGAS00001000127); a full list of the investigators who contributed to the generation of the data is available from http://www.UK10K.org. Access to data generated by the UK10K_Rare_Coloboma (EGA Study ID: EGAS00001000127) project of the UK10K Consortium study (http://www.UK10K.org) was obtained through a Data Access Agreement. | Yes | - | Yes | - | - | Yes | - | - | - | Yes | - | Yes | - | - | Yes | - | - | - |
| 2 | PG2 | In the present study, the informed consents of participants have not been conducted and given, since 1) the privacy and personal identity information of all participants were protected, i.e., all the data were analyzed anonymously, 2) all the blood samples were not and will not be used for any other purpose and 3) the waiver of informed consent did not and will not have adverse effect on the rights and health of the participants. This study was approved by the Institute Research Medical Ethics Committee at Sun Yat-Sen University Cancer Center. | - | Yes | - | Yes | - | Yes | - | - | - | Yes | - | Yes | - | Yes | - | - | Yes | - |
| 3 | PG3 | All DNA samples were collected and genotyped following signed and informed written consent from a parent or guardian. Ethics approval for all procedures was obtained from both LSHTM(#2087) and the Tanzanian National Institute of Medical Research (NIMR/HQ/R.8a/Vol.IX/392). | Yes | - | Yes | - | - | Yes | - | - | - | Yes | - | Yes | - | - | Yes | - | - | - |
| 4 | PG4 | Crohn’s disease patients for the sequencing experiment (n = 474) were recruited from specialist IBD clinics in London and Newcastle [37] after informed consent and ethical review (REC 05/Q0502/127). Population controls for sequencing (n = 480) were obtained from the 1958 British Birth Cohort [38]. | Yes | - | - | Yes | - | Yes | - | - | - | Yes | - | Yes | - | - | Yes | - | - | - |
| 5 | PG5 | The SPARTAC trial was approved by the following authorities: Medicines and Healthcare products Regulatory Agency (UK), Ministry of Health (Brazil), Irish Medicines Board (Ireland), Medicines Control Council (South Africa), and The Uganda National Council for Science and Technology (Uganda). It was also approved by the following ethics committees in the participating countries: Central London Research Ethics Committee (UK), Hospital Universitrio Clementino Fraga Filho Ethics in Research Committee (Brazil), Clinical Research and Ethics Committee of Hospital Clinic in the province of Barcelona, Spain, The Adelaide and Meath Hospital Research Ethics Committee (Ireland), University ofWitwatersrand Human Research Ethics Committee, University of Kwazulu-Natal Research Ethics Committee and University of Cape Town Research Ethics Committee (South Africa), Uganda Virus Research Institute Science and ethics committee (Uganda), The Prince Charles Hospital Human Research Ethics Committee and St Vincent’s Hospital Human Research Ethics Committee (Australia), and the National Institute for Infectious Diseases Lazzaro Spallanzani, Institute Hospital and the Medical Research Ethics Committee, and the ethical committee Of the Central Foundation of San Raffaele, MonteTabor (Italy). All participants signed a written informed consent.All participants gave written informed consent. The trial was approved by research ethics committees in each country. | Yes | - | - | Yes | - | Yes | - | - | - | Yes | - | Yes | - | - | Yes | - | - | - |
| 6 | PG6 | The study design was approved by the ethics committee of Azienda Sanitaria Locale Napoli 1. The study was conducted according to the criteria set by the declaration of Helsinki and each subject signed an informed consent before participating to the study. | Yes | - | - | Yes | - | Yes | - | - | - | Yes | - | Yes | - | - | Yes | - | - | - |
| 7 | PG7 | This study was approved by the Institutional Review Boards of both Yale University and the Broad Institute. | - | Yes | - | - | - | - | - | - | - | Yes | - | Yes | - | - | Yes | - | - | - |
| 8 | PG8 | The study protocol was approved by the medical ethics board of the Erasmus MC Rotterdam, the Netherlands. The study included only adults and written informed consents were provided by all the subjects participated in the study | Yes | - | - | Yes | - | Yes | - | - | - | Yes | - | Yes | - | - | Yes | - | - | - |
| 9 | PG9 | Immunohistochemistry staining of paraffin embedded human lung tissue of de-identified patients was carried out with the IRB approval (HS#12-00171) from Mount Sinai Hospital.[…]The paraffin sections of human lung tissues were provided by Histology Shared Resource Facility of Mount Sinai Hospital with the IRB approval. | - | Yes | - | - | - | - | - | - | - | Yes | - | Yes | - | - | Yes | - | - | - |
| 10 | PG10 | The cases reported here (18 males, 23 females) with moderate (n=12) or severe (n=29) ID were recruited at the Sainte-Justine Hospital (Montreal, Canada), after the approval of the ethics committee, and informed consent was obtained from each participant or legal guardian. | Yes | - | Yes | - | - | Yes | - | - | - | Yes | - | Yes | - | - | Yes | - | - | - |
| 11 | PG11 | They refer to other papers, but use biomaterial from those studies | - | Yes | - | - | - | - | - | - | - | - | Yes | - | - | - | - | - | - | - |
| 12 | PG12 | 40 lung tumor tissue samples were provided from the Department of Pathology, Ohio State University. All human tissues were obtained according to a protocol approved by the Ohio State Institutional Review Board. | - | Yes | - | - | - | - | - | - | - | Yes | - | Yes | - | - | Yes | - | - | - |
| 13 | PG13 | Approval for the research study was obtained from the IWK Health Centre research ethics board (project approval number 1005367). Informed consent was obtained from individuals or their guardians for all samples used in this study. DNA was obtained from blood, saliva or FFPE samples using standard methods | Yes | - | Yes | - | - | Yes | - | - | - | Yes | - | Yes | - | - | Yes | - | - | - |
| 14 | PG14 | All studies were approved by their local ethics committees; all patients gave informed consent. | Yes | - | - | Yes | - | Yes | - | - | - | Yes | - | - | Yes | - | Yes | - | - | - |
| 15 | PG15 | The study was approved by the Research Ethics Committee of the National Centre of Medical Genetics of Cuba. Each individual in this study gave written informed consent prior to the interview, physical examination and blood sample collection. | Yes | - | Yes | - | - | Yes | - | - | - | Yes | - | Yes | - | - | Yes | - | - | - |
| 16 | PG16 | Ethical approval for the Belem Family Study was obtained originally from the local ethics committee at the Instituto Evandro Chagas, Belem, Para, Brazil. Approval for continued use of the Belem Family Study samples, and for collection and use of the samples from Natal, has been granted from the local Institutional Review Board at the Universidade Federal do Rio Grande do Norte (CEP-UFRN 94–2004), nationally from the Comissatica em Pesquisa (CONEP: 11019), and from the Nacional de EMinisterios Cencia e Tecnologia for approval to ship samples out of Brazil (portaria 617; 28 September 2005). Informed written consent for sample collection was obtained from adults, and from parents of children v18 years old | Yes | - | Yes | - | - | Yes | - | - | - | Yes | - | Yes | - | Yes | - | - | - | Yes |
| 17 | PG17 | All patients were prospectively enrolled using protocols approved by the appropriate IRBs. | - | Yes | - | - | - | - | - | - | - | Yes | - | - | Yes | - | Yes | - | - | - |
| 18 | PG18 | All subjects provided written informed consent authorizing whole genome sequencing on a protocol approved by the Washington University Office of Human Research Protection | Yes | - | Yes | - | - | Yes | - | - | - | Yes | - | Yes | - | - | Yes | - | - | - |
| 19 | PG19 | Ethical approval for the study was obtained from the ALSPAC Ethics and Law Committee and the Local Research Ethics Committees. | - | Yes | - | - | - | - | - | - | - | Yes | - | Yes | - | - | Yes | - | - | - |
| 20 | PG20 | The Institutional Review Board at the University of Utah approved this study. Written informed consent was obtained from all participants for their tissues to be utilized for this work. | Yes | - | Yes | - | - | Yes | - | - | - | Yes | - | Yes | - | - | Yes | - | - | - |
| 21 | PG21 | Informed consent was obtained from all participants. Study approval was provided by the University of Alberta Hospital Health Research Ethics Board and the Ethics Committee of the IRCCS Oasi Maria SS Onlus, Troina, Italy. | Yes | - | - | Yes | - | Yes | - | - | - | Yes | - | Yes | - | - | Yes | - | - | - |
| 22 | PG22 | All specimens for sequencing were obtained from patients with appropriate consent from the local institutional review board in accordance with the Children’s Oncology Group and the National Cancer Institute. Clinical samples were obtained from collaborations with the Cooperative Human Tissue Network, the Children’s Hospital of Westmead, Australia, the Children’s Oncology Group, and the National Institutes ofHealth Clinical Center. | - | Yes | - | - | - | - | - | - | - | Yes | - | - | Yes | - | Yes | - | - | - |
| 23 | PG23 | All participants provided written informed consent and studies were approved by local Research Ethics Committees and/or Institutional Review Boards. | Yes | - | - | Yes | - | Yes | - | - | - | Yes | - | - | Yes | - | Yes | - | - | - |
| 24 | PG24 | All research was approved by our Institutional Review Board, and informed consent was obtained from each volunteer.We enrolled 225 healthy volunteers (134 females, 91 males) of non-Hispanic Caucasian descent that proved informed consent through the Phenogenetics Project at Brigham and Women’s Hospital. | Yes | - | - | Yes | - | Yes | - | - | - | Yes | - | Yes | - | - | Yes | - | - | - |
| 25 | PG25 | Ethical approval for the GLACIER Study was obtained from the Regional Ethical Review Board in Umea, Sweden. The Ethics Committee at Lund University approved the MDC study. All participants provided written informed consent as part of the VHU. (note: VHU = Väserbotten Health Survey, prospective population based cohort study) | Yes | - | Yes | - | Yes | - | Yes | - | - | Yes | - | Yes | - | Yes | - | - | Yes | - |
| 26 | PG26 | Saliva samples were collected in Yemen in 2007 with informed consent under Western IRB approval, Olympia, WA. Subsequent analysis of anonymized SNP data was approved by the Lehman College IRB. | Yes | - | - | Yes | - | Yes | - | - | - | Yes | - | Yes | - | Yes | - | - | - | Yes |
| 27 | PG27 | This study was approved by the Ethics Committee of the Center for Public Health Research (CSISP), Valencia, Spain. All women participating in the study read and signed forms of informed consent specifically approved for this project by the Ethics Committee. | Yes | - | Yes | - | - | Yes | - | - | - | Yes | - | Yes | - | - | Yes | - | - | - |
| 28 | PG28 | All primary tissue samples and blood for this project were obtained from adult cadaveric organ transplant donors referred to the Eastern Organ Donation Services Team (part of NHS Blood and Transplant). Ethics approval was obtained from the local Research Ethics Committee (REC No. 09/H306/73). | - | Yes | - | - | - | - | - | - | - | Yes | - | Yes | - | - | Yes | - | - | - |
| 29 | PG29 | All patient recruitment and sample collection was performed with the appropriate human subjects protocol approval from the Institutional Review Board at the Albert Einstein College of Medicine. All adult subjects provided written informed consent and a parent or guardian of any child participant provided written informed consent on their behalf. | Yes | - | Yes | - | - | Yes | - | - | - | Yes | - | Yes | - | - | Yes | - | - | - |
| 30 | PG30 | This research was approved by the Institutional Review Board of Duke University Medical Center and adheres to the tenets of the Declaration of Helsinki | - | Yes | - | - | - | - | - | - | - | Yes | - | Yes | - | - | Yes | - | - | - |
| 31 | NE1 | The protocol was approved by the institutional review board at each center, and the study was conducted in accordance with the Declaration of Helsinki and the International Conference on Harmonisation Guidelines for Good Clinical Practice. All the patients provided written informed consent before study entry | Yes | - | - | Yes | - | Yes | - | - | - | Yes | - | - | Yes | - | Yes | - | - | - |
| 32 | NE2 | The institutional review board at each of the participating institutions approved our studies. We studied the anonymized samples from international acromegaly cohorts with approval from the National Institutes of Health Intramural Office for Human Research Protections. Written informed consent was obtained from all adult patients and parents or guardians of children with early-onset gigantism. | Yes | - | Yes | - | - | Yes | - | - | - | Yes | - | Yes | - | - | Yes | - | - | - |
| 33 | NE3 | All procedures were approved by the relevant ethics committees, and written informed consent was obtained from all participants. | Yes | - | - | Yes | - | Yes | - | - | - | Yes | - | - | Yes | - | Yes | - | - | - |
| 34 | NE4 | The protocols for these studies were approved by the ethics committees at all involved institutions; written informed consent was obtained from all participants.The remaining 1,381 individuals were additional subjects in the Jackson Heart Study (JHS), a large population based cohort of AfricanAmericans in Jackson, Mississippi, who had given consent for genetic testing but were not in any previously sequenced cohorts. | Yes | - | Yes | - | Yes | - | Yes | - | - | Yes | - | Yes | - | Yes | - | - | Yes | - |
| 35 | NE5 | Primary tumor samples and matched normal peripheral-blood specimens were obtained after the patients had provided written informed consent. Primary tumor samples and matched normal specimens (peripheral blood) were obtained with written informed consent per approved institutional review board (IRB) protocols. | Yes | - | - | Yes | - | Yes | - | - | - | Yes | - | - | Yes | - | Yes | - | - | - |
| 36 | NE6 | Written informed consent was obtained from all the participants. The protocol for this study was approved by the institutional review board at the University of Texas Southwestern Medical Center. | Yes | - | - | Yes | - | Yes | - | - | - | Yes | - | Yes | - | - | Yes | - | - | - |
| 37 | NE7 | All study participants gave written informed consent, and the study was conducted in accordance with the provisions of the Declaration of Helsinki. | Yes | - | - | Yes | - | Yes | - | - | - | - | Yes | - | - | - | - | - | - | - |
| 38 | NE8 | Written informed consent for targeted whole-exome sequencing and Sanger sequencing was obtained from the parents of the index patient for themselves, the index patient, and her two siblings. Sanger sequencing of candidate genes in the cohort of patients with a suspected ketolytic defect and blood collection for functional studies were performed as a part of the diagnostic process (approved by the medical ethics committee of the University Medical Center Utrecht). | Yes | - | Yes | - | - | Yes | - | - | - | Yes | - | Yes | - | - | Yes | - | - | - |
| 39 | NE9 | We conducted this study using data and DNA samples from 16 case–control studies and cohort studies. All study participants provided written informed consent for genetic studies. The institutional review boards at the Broad Institute and each participating site approved the study protocols. | Yes | - | Yes | - | Yes | - | Yes | - | - | Yes | - | Yes | - | Yes | - | - | Yes | - |
| 40 | NE10 | The study was conducted in accordance with International Conference on Harmonisation Guidelines for Good Clinical Practice, applicable regulations, and guidelines governing clinical-study conduct that have their origin in the Declaration of Helsinki. All patients provided written informed consent. The study was designed by the sponsor (AbbVie); the investigators and the sponsor jointly conducted the study and gathered the data. The sponsor conducted the data analyses. All authors signed a confidentiality agreement with the sponsor. | Yes | - | - | Yes | - | Yes | - | - | - | - | Yes | - | - | - | - | - | - | - |
| 41 | NE11 | Approval and a waiver from the need to provide written informed consent were obtained from the ethics review committees for the Guinean government and the WHO. | Yes | - | Yes | - | - | Yes | - | - | - | Yes | - | Yes | - | - | Yes | - | - | - |
| 42 | NE12 | The institutional review board at Tulane University, the ethics committee at Harvard University, and the Sierra Leone Ethics and Scientific Review Committee approved this project. These committees waived the requirement to obtain informed consent during the West African Ebola outbreak. All clinical samples and data were collected for routine patient care and for public health interventions. The institutional biosafety committees at Tulane and Harvard and the Sierra Leone Ethics and Scientific Review Committee reviewed and approved biosafety protocols for this study. | Yes | - | Yes | - | - | Yes | - | - | - | Yes | - | Yes | - | - | Yes | - | - | - |
| 43 | NE13 | The study was conducted in accordance with the Declaration of Helsinki and adhered to Good Clinical Practice guidelines. Approval for the protocol was obtained from the local ethics committee for each participating site. All patients provided written informed consent, including a separate, specific signature consenting to blood sampling and specimen donation for translational analyses. | Yes | - | Yes | - | - | Yes | - | - | - | Yes | - | - | Yes | - | Yes | - | - | - |
| 44 | NE14 | Written informed consent for participation was obtained from patients or their guardians according to the Declaration of Helsinki and protocols were approved by the institutional review boards of the Children’s Hospital of Philadelphia and the Hospital of the University of Pennsylvania, respectively. | Yes | - | Yes | - | - | Yes | - | - | - | Yes | - | Yes | - | - | Yes | - | - | - |
| 45 | NE15 | As in previous EVD outbreaks, blood samples were collected, with oral consent, either at the homes of patients or in hospital isolation wards, by a team that included staff members of the Ministry of Health in the DRC and the WHO. | Yes | - | Yes | - | - | Yes | - | - | - | - | Yes | - | - | - | - | - | - | - |
| 46 | NE16 | The parents of 1343 children provided written informed consent for the study. The study was approved by the local medical ethical committees of the participating centers and complied with the Good Clinical Practices (ICH-GCP) regulations. An independent ethical advisor, Professor Moshe Berant (Helsinki Committee, Rambam Health Care Campus, Haifa, Israel) was appointed to provide guidance on ethical issues and decisions throughout the research period. An ethical sub-committee, chaired by the ethical advisor, monitored the project’s management of documentation and reporting, and its safety issues. | Yes | - | Yes | - | - | Yes | - | - | - | Yes | - | Yes | - | - | Yes | - | - | - |
| 47 | NE17 | The study protocol was approved by the institutional review board at each participating center. Written informed consent was obtained from the parents or guardians of the children | Yes | - | Yes | - | - | Yes | - | - | - | Yes | - | Yes | - | - | Yes | - | - | - |
| 48 | NE18 | After the approvals from ethics committees and data-protection agencies were obtained, patients in 32 general ICUs in Denmark, Sweden, Norway, and Finland underwent screening and randomization between December 3, 2011, and December 26, 2013. Written informed consent was obtained from all the patients or their legal surrogates before or after enrollment. In all cases, consent was obtained from the patient when possible. If consent was withdrawn or not granted, we asked the patient or surrogate for permission to continue registration of trial data and to use these data in the analyses | Yes | - | Yes | - | - | Yes | - | - | - | Yes | - | Yes | - | - | Yes | - | - | - |
| 49 | NE19 | The study was approved by the institutional review board or ethics committee at each participating institution and was conducted in accordance with the provisions of the Declaration of Helsinki and the International Conference on Harmonisation guidelines for Good Clinical Practice. All the patients provided written informed consent. An independent data and safety monitoring committee conducted regular review and evaluation of the safety data. | Yes | - | - | Yes | - | Yes | - | - | - | Yes | - | - | Yes | - | Yes | - | - | - |
| 50 | NE20 | The protocol was approved by the institutional review board or human research ethics committee at each participating center and complied with country-specific regulatory requirements. The study was conducted in accordance with the provisions of the Declaration of Helsinki and Good Clinical Practice guidelines. Written informed consent was obtained from all study patients. | Yes | - | - | Yes | - | Yes | - | - | - | Yes | - | - | Yes | - | Yes | - | - | - |
| 51 | NE21 | The protocol, which is available at NEJM.org, was approved by the institutional review board or independent ethics committee at each site and complied with the International Ethical Guidelines for Biomedical Research Involving Human Subjects, Good Clinical Practice guidelines, the Declaration of Helsinki, and local laws. All patients provided written informed consent. | Yes | - | - | Yes | - | Yes | - | - | - | Yes | - | - | Yes | - | Yes | - | - | - |
| 52 | NE22 | Samples were obtained from patients enrolled under clinical-trial protocols of St. Jude Children’s Research Hospital, the Children’s Oncology Group, the Eastern Cooperative Oncology Group, the Alliance for Clinical Trials in Oncology (Cancer and Leukemia Group B), and M.D. Anderson Cancer Center. The details of the treatment protocols are provided in Supplementary Appendix 2. Patients, parents, or guardians gave written informed consent for sample collection and research, with assent provided by older children and adolescents. The study was approved by the St. Jude Institutional Review Board. | Yes | - | Yes | - | - | Yes | - | - | - | Yes | - | Yes | - | - | Yes | - | - | - |
| 53 | NE23 | The studies were approved by the Human Research Ethics Committee of the University of the Witwatersrand and were conducted in accordance with Good Clinical Practice guidelines. Written informed consent was obtained from all participants. | Yes | - | - | Yes | - | Yes | - | - | - | Yes | - | Yes | - | - | Yes | - | - | - |
| 54 | NE24 | The study was approved by the institutional review board at Johns Hopkins University. All enrolled patients provided written informed consent.qRT-PCR analysis for AR-V7 was performed on fresh metastatic tumor biopsies or autopsy specimens) from a subset of patients who consented to this. | Yes | - | Yes | - | - | Yes | - | - | - | Yes | - | Yes | - | - | Yes | - | - | - |
| 55 | NE25 | All household contacts of index patients (who were defined as persons living in the same house, regardless of the degree of relation to the index patient) were obliged to participate in the investigation per decree of the Ministry of Health as part of a public health response | Yes | - | Yes | - | - | Yes | - | - | - | - | Yes | - | - | - | - | - | - | - |
| 56 | NE26 | The study protocol was approved by the institutional review board of the Johns Hopkins University School of Medicine, and written informed consent was obtained from each patient, in accordance with the Declaration of Helsinki. | Yes | - | - | Yes | - | Yes | - | - | - | Yes | - | Yes | - | - | Yes | - | - | - |
| 57 | NE27 | The study was approved by the institutional review boards of Boston Children’s Hospital and Beth Israel Deaconess Medical Center, Boston. Written informed consent was obtained from all participants or their parents or guardians. | Yes | - | Yes | - | - | Yes | - | - | - | Yes | - | Yes | - | - | Yes | - | - | - |
| 58 | NE28 | The study was approved by the National Research Ethics Service London — City and East (Bristol, United Kingdom) and conducted in accordance with International Conference on Harmonisation Good Clinical Practice guidelines, applicable regional and local regulations, the Declaration of Helsinki (1996), and the study protocol. Participants provided written informed consent before study-specific procedures were performed. | Yes | - | - | Yes | - | Yes | - | - | - | Yes | - | Yes | - | - | Yes | - | - | - |
| 59 | NE29 | The study was approved by the research ethics committee at each participating center and at Hamilton Health Sciences. All the study participants provided written informed consent. | Yes | - | - | Yes | - | Yes | - | - | - | Yes | - | Yes | - | - | Yes | - | - | - |
| 60 | NE30 | The study was approved by the ethics committees at all participating centers and at Hamilton Health Sciences, Hamilton, Ontario, Canada. All participants provided written informed consent. | Yes | - | - | Yes | - | Yes | - | - | - | Yes | - | Yes | - | - | Yes | - | - | - |
| 61 | NG1 | Patients were enrolled and sampled according to standard local practice in approved human subject protocols at the University of California. Sampling was performed on both parents and all available genetically informative siblings to include affected and affected family members, as well as extended family members if appropriate, upon informed consent approval and consistent with institutional review board (IRB) guidelines. Blood and/or saliva was collected for all consenting, potentially informative family members; | Yes | - | Yes | - | - | Yes | - | - | - | Yes | - | - | Yes | - | Yes | - | - | - |
| 62 | NG2 | All tumor samples were collected at the time of surgery after informed consent was obtained. This study was approved by the ethics committees at all the participating institutes. | Yes | - | - | Yes | - | Yes | - | - | - | Yes | - |  | Yes | - | Yes | - | - | - |
| 63 | NG3 | This study was approved by the institutional review board at the University of Texas Southwestern Medical Center. Written informed consent was obtained from all participants. | Yes | - | - | Yes | - | Yes | - | - | - | Yes | - | Yes | - | - | Yes | - | - | - |
| 64 | NG4 | A series of 243 liver tumor samples and their non-tumor counterparts were collected from patients surgically treated in Europe: 193 cases from France (Créteil and Bordeaux), 9 cases from Spain (Barcelona) and 41 cases from Italy (Milan). The study was approved by institutional review board committees (CCPRB Paris Saint-Louis, 1997, 2004 and 2010, approval number 01-037; Bordeaux, 2010-A00498-31). Written informed consent was obtained in accordance with French legislation. All samples were immediately frozen in liquid nitrogen and stored at −80 °C. | Yes | - | - | Yes | - | Yes | - | - | - | Yes | - | Yes | - | - | Yes | - | - | - |
| 65 | NG5 | This study is based on whole-genome sequence data from the white blood cells of 2,636 Icelanders participating in various disease projects at deCODE Genetics (Supplementary Tables 1 and 2). In addition, a total of 104,220 Icelanders have been genotyped using Illumina SNP chips (Supplementary Table 6). All participating individuals, or their guardians, gave their informed consent before blood samples were drawn. The family history of participants donating blood was incorporated into the study by including the phenotypes of first-and second-degree relatives and integrating over their possible genotypes. This integration is performed without the genotypes being stored. All sample identifiers were encrypted in accordance with the regulations of the Icelandic Data Protection Authority. Approval for these studies was provided by the National Bioethics Committee and the Icelandic Data Protection Authority. | Yes | - | Yes | - | Yes | - | Yes | - | - | Yes | - | Yes | - | Yes | - | Yes | - | - |
| 66 | NG6 | This study is based on whole-genome sequencing data from the whole blood of 2,636 Icelanders participating in various disease projects at deCODE Genetics28. Approval for these studies was provided by the National Bioethics Committee and the Icelandic Data Protection Authority. The appropriate informed consent was obtained from all participants before blood samples were drawn, and all sample identifiers were encrypted in accordance with the regulations of the Icelandic Data Protection Committee. | Yes | - | Yes | - | Yes | - | Yes | - | - | Yes | - | Yes | - | Yes | - | Yes | - | - |
| 67 | NG7 | Patient tissue acquisition. All patients in the original cohort (family 1) were recruited at the Hematology Clinic at the Children’s Hospital of Michigan. The study received institutional review board approval from the University of Colorado Anschutz Medical Campus, and informed consent was obtained for all participants. Patients in families 2 and 3 were recruited in clinical centers in the Czech Republic and Italy as part of a European consortium focused on inherited platelet disorders. The institutional review board of the IRCCS Policlinico San Matteo Foundation of Pavia, Italy, approved the study protocol. Informed consent was obtained from all patients in the study. Studies were performed in accordance with the Declaration of Helsinki. Genomic DNA was extracted from whole blood using the Gentra PureGene DNA Extraction kit (Qiagen). Platelets were purified from whole blood and negatively selected using CD45+ MACS MicroBeads (Miltenyi Biotec). Bone marrow aspirates were obtained at diagnosis, and permission was obtained from families to use images. | Yes | - | - | Yes | - | Yes | - | - | - | Yes | - | Yes | - | - | Yes | - | - | - |
| 68 | NG8 | The Icelandic study was approved by the Data Protection Authority and the National Bioethics Committee, and the non-Icelandic studies were approved by local ethics boards. Participants giving samples also gave written, informed consent. | Yes | - | - | Yes | - | Yes | - | - | - | Yes | - | Yes | - | - | Yes | - | - | - |
| 69 | NG9 | All participating individuals, or their guardians, gave their informed consent before blood samples were drawn. All sample identifiers were encrypted in accordance with the regulations of the Icelandic Data Protection Authority. Approval for these studies was provided by the National Bioethics Committee and the Icelandic Data Protection Authority. | Yes | - | Yes | - | - | Yes | - | - | - | Yes | - | Yes | - | - | Yes | - | - | - |
| 70 | NG10 | The medical ethical review committees of all participating study centers approved this study. Informed consent was obtained from all subjects. | Yes | - | - | Yes | - | Yes | - | - | - | Yes | - | - | Yes | - | Yes | - | - | - |
| 71 | NG11 | Blood samples from all participants were collected and studied with written informed consent according to the Declaration of Helsinki and with approvals from the local ethics committees in Russia (St. Petersburg and Samara) and the UK (Human Biological Resource Ethics Committee of the University of Cambridge and the National Research Ethics Service,Cambridgeshire 1 REC, 10/H0304/71). | Yes | - | - | Yes | - | Yes | - | - | - | Yes | - | Yes | - | - | Yes | - | - | - |
| 72 | NG12 | Informed consent was obtained from the patients or their legal guardians prior to analysis. The study was approved by the ethics committees of the University of Antwerp, Belgium; University of Bern, Switzerland; and University of Kiel, Germany. | Yes | - | Yes | - | - | Yes | - | - | - | Yes | - | Yes | - | - | Yes | - | - | - |
| 73 | NG13 | All participating studies were approved by their appropriate ethics review boards, and all subjects provided informed consent. | Yes | - | - | Yes | - | Yes | - | - | - | Yes | - | - | Yes | - | Yes | - | - | - |
| 74 | NG14 | All DNA samples were collected after approval from relevant institutional research ethics committees. Review boards of all contributing institutions approved all protocols and informed consent for sharing of data and sample collection; appropriate informed consent was obtained from all subjects and families. | Yes | - | - | Yes | Yes | - | Yes | - | - | Yes | - | - | Yes | Yes | - | - | - | Yes |
| 75 | NG15 | All individuals enrolled in the study were evaluated by clinical geneticists experienced in the diagnosis of CdLS. All patients and family members were enrolled in the study under an institutional review board–approved protocol of informed consent at the Children’s Hospital of Philadelphia. | Yes | - | - | Yes |  | Yes | - | - | - | Yes | - | Yes | - | - | Yes | - | - | - |
| 76 | NG16 | Tumor and germline samples were obtained with informed consent using a protocol approved by the St. Jude Children’s Research Hospital institutional review board. The study was approved by the institutional review boards of St. Jude Children’s Research Hospital and Washington University. | Yes | - | - | Yes | - | Yes | - | - | - | Yes | - | Yes | - | - | Yes | - | - | - |
| 77 | NG17 | Samples for analysis were collected from prostatectomy patients at Addenbrooke’s Hospital (see Supplementary Table 2). The study was approved by the Trent Multicentre Research Ethics Committee. Informed consent was obtained for all patients. | Yes | - | - | Yes | - | Yes | - | - | - | Yes | - | Yes | - | - | Yes | - | - | - |
| 78 | NG18 | All cases and controls were enrolled in the study following informed consent and ethical approval from the relevant national and regional institutional review boards for each sample collection. | Yes | - | - | Yes | - | Yes | - | - | - | Yes | - | - | Yes | - | Yes | - | - | - |
| 79 | NG19 | The ethics committee of CHU Sainte-Justine approved the study protocol, and informed consent was obtained from all subjects. | Yes | - | - | Yes | - | Yes | - | - | - | Yes | - | Yes | - | - | Yes | - | - | - |
| 80 | NG20 | The study protocol was reviewed by the Boston University Medical Center Institutional Review Board, and all participants gave written informed consent. | Yes | - | - | Yes | - | Yes | - | - | - | Yes | - | - | Yes | - | Yes | - | - | - |
| 81 | NG21 | This study was approved by the St. Jude Children’s Research Hospital institutional review board, and informed consent was obtained from all patients, parents or legal guardians as appropriate. | Yes | - | Yes | - | - | Yes | - | - | - | Yes | - | Yes | - | - | Yes | - | - | - |
| 82 | NG22 | This study employed deidentified excess tissue specimens collected in the course of routine clinical care and was approved by the local institutional review board (IRB). | - | Yes | - | Yes | - | Yes | - | - | - | Yes | - | - | Yes | - | Yes | - | - | - |
| 83 | NG23 | The cases and controls were recruited using uniform criteria with written informed consent and were matched by ancestry and geographical area. The study was approved by the institutional review board (IRB) committees at the Shandong Provincial Institute of Dermatology and Venereology, Shandong Academy of Medical Science. | Yes | - | - | Yes | - | Yes | - | - | - | Yes | - | Yes | - | - | Yes | - | - | - |
| 84 | NG24 | Following Institutional Research Ethics Board approval, all data were centralized in the Division of Haematology/Oncology at The Hospital for Sick Children (SickKids) and the Familial Gastrointestinal Cancer Registry (FGICR) at the Zane Cohen Centre for Digestive Diseases at Mount Sinai Hospital, in Toronto, Canada. Consent forms were obtained from the parents or guardians, or from the patients, where applicable. | Yes | - | Yes | - | - | Yes | - | - | - | Yes | - | Yes | - | - | Yes | - | - | - |
| 85 | NG25 | All samples in this study were derived from blood samples obtained from patients with P. falciparum malaria, collected with informed consent from the patient or a parent or guardian. At each location, sample collection was approved by the appropriate local ethics committee: Ethical Committee, Hospital for Tropical Diseases, Ho Chi Minh City, Vietnam; Ethics Committee for Biomedical Research of the Ministry of Health, Institute of Malariology-Parasitology-Entomology, Ho Chi Minh City, Vietnam; National Ethics Committee for Health Research, Ministry of Health, Phnom Penh, Cambodia; Ethics Committee, Faculty of Tropical Medicine, Mahidol University, Bangkok, Thailand; Tak Province Community Ethics Advisory Board (T-CAB), Tak, Thailand; Government of the Republic of the Union of Myanmar, Ministry of Health, Department of Medical Research (lower Myanmar); National Ethics Committee for Health Research, Ministry of Health, Lao Peoples’ Democratic Republic; National Research Ethics Committee, Bangladesh Medical Research Council; Comité d’Ethique, Ecole de Santé Publique, Université de Kinshasa, Ministère de l’Enseignement Superieur, Universitaire et Recherche Scientifique, Democratic Republic of the Congo; Ethical Review Committee, University of Ilorin Teaching Hospital, Ilorin, Nigeria; Navrongo Health Research Centre Institutional Review Board, Navrongo, Ghana; Institutional Review Board, National Institute of Allergy and Infectious Diseases, Bethesda, Maryland, USA; Ethics Review Committee, World Health Organization, Geneva, Switzerland; and Oxford Tropical Research Ethics Committee (OxTREC), Oxford, UK. | Yes | - | - | Yes | - | Yes | - | - | - | Yes | - | Yes | - | - | Yes | - | - | - |
| 86 | NG26 | All subjects included in this analysis were of European descent and provided written informed consent as well as data and blood samples under ethically approved protocols. | Yes | - | - | Yes | - | Yes | - | - | - | Yes | - | - | Yes | - | Yes | - | - | - |
| 87 | NG27 | Subjects provided written informed consent in accordance with protocols approved by the institutional review boards of the Fred Hutchinson Cancer Research Center and Seattle Children’s Hospital for family A and the University of Chicago for families B and C. | Yes | - | - | Yes | - | Yes | - | - | - | Yes | - | Yes | - | - | Yes | - |  | - |
| 88 | NG28 | Written informed consent was obtained from each patient, and the study was approved by the institution’s ethics committee. | Yes | - | - | Yes | - | Yes | - | - | - | Yes | - | - | Yes | - | Yes | - | - | - |
| 89 | NG29 | All participating centers received approval from their local and national institutional review boards, and informed consent was obtained from all participants in the study. | Yes | - | - | Yes | - | Yes | - | - | - | Yes | - | - | Yes | - | Yes | - | - | - |
| 90 | NG30 | Participation in this study by patients and their relatives along with skin biopsy donations and informed consent procedures were approved by the ethics committees of the Genomic and Genetic Disorder Biobank (Casa Sollievo della Sofferenza, San Giovanni Rotondo, Italy) and the University of Perugia (Azienda Ospedaliera–Universitaria ‘Santa Maria della Misericordia’, Perugia, Italy). | Yes | - | - | Yes | - | Yes | - | - | - | Yes | - | Yes | - | - | Yes | - | - | - |
| 91 | NM1 | The study was performed and all human tissues were obtained with informed consent in accordance with protocols approved by Severance Hospital and KAIST Institutional Review Board and Committee on Human Research. | Yes | - | - | Yes | - | Yes | - | - | - | Yes | - | Yes | - | - | Yes | - | - | - |
| 92 | NM2 | Human islets were obtained from the Integrated Islet Distribution Program (IIDP) at City of Hope and from the San Raffaele Scientific Institute, Milan, Italy. All studies and protocols were approved by the respective ethics committees (ethics committee of the Medical Faculty, Heinrich Heine University Düsseldorf, study number 3921; ethics committee of the Instituto Scientifico Ospedale San Raffaele; and ethics committees of the IIDP centers). | - | Yes | - | - | - | - | - | - | - | Yes | - | Yes | - | - | Yes | - | - | - |
| 93 | NM3 | Hippocampal samples from adult humans with Down syndrome and age- and sex-matched controls were obtained from the Brain and Tissue Bank for Developmental Disorders at the University of Maryland, Baltimore, MD. | - | Yes | - | - | - | - | - | - | - | - | Yes | - | - | - | - | - | - | - |
| 94 | NM4 | The study protocol was approved by the ethics committee Ethikkommission St. Gallen. Written informed consent was obtained from each patient before tissue collection. | Yes | - | - | Yes | - | Yes | - | - | - | Yes | - | Yes | - | - | Yes |  | - |  |
| 95 | NM5 | From a cohort of Canadian ASD families, we selected 85 unrelated families with at least two children with ASD. Both parents and two children with ASD were recruited, with selection based on availability of genomic DNA from whole blood and completeness of phenotype information. We recruited additional siblings and members of the extended family across generations whenever possible. We obtained informed consent from all participants, as approved by the Research Ethics Boards at The Hospital for Sick Children, McMaster University and Memorial Hospital. | Yes | - | - | Yes | - | Yes | - | - | - | Yes | - | Yes | - | - | Yes | - | - | - |
| 96 | NM6 | We obtained nucleated bone marrow cells (BMCs) from patients with acute MI enrolled in the BOOST-2 trial. The BOOST-2 trial is registered at Current Controlled Trials (International Standard Randomized Controlled Trial Number: ISRCTN17457407). See http://www.isrctn.com/ ISRCTN17457407 for the study protocol. The Ethics Committee of Hannover Medical School approved the study. All patients provided written informed consent to participate in BOOST-2 and to donate an aliquot of their bone marrow for use in the present study. | Yes | - | Yes | - | Yes | - | - | Yes | - | Yes | - | Yes | - | - | Yes | - | - | - |
| 97 | NM7 | PBMCs were isolated from donated human whole blood after informed consent had been obtained (Gulf Coast Regional Blood Center, Houston, TX) | Yes | - | - | Yes | - | Yes | - | - | - | - | Yes | - | - | - | - | - | - | - |
| 98 | NM8 | Approval to obtain whole blood samples from healthy volunteers was obtained from the Institutional Review Board of Brigham and Women’s Hospital, and informed consent was approved according to the Declaration of Helsinki. | Yes | - | - | Yes | - | Yes | - | - | - | Yes | - | Yes | - | - | Yes | - | - | - |
| 99 | NM9 | All participants provided informed written consent for tumor analysis, and all studies were approved by the Dana-Farber Cancer Institute Institutional Review Board. | Yes | - | - | Yes | - | Yes | - | - | - | Yes | - | Yes | - | - | Yes | - | - | - |
| 100 | NM10 | Lymph node specimens from cohort 1 (45 human subjects) were obtained from the archives of the Department of Pathology of the Technical University of Munich; specimens from cohort 2 (81 human subjects) were from the archives of the lymph node registry of the German Low Grade Lymphoma Study Group (GLSG); and specimens from cohort 3 (20 human subjects) were from the archives of the Department of Pathology of the University of Würzburg .All human specimens were processed with informed consent in compliance with the institutional review board at the Faculty of Medicine of the Technical University of Munich. | Yes | - | - | Yes | - | Yes | - | - | - | Yes | - | Yes | - | - | Yes | - | - | - |
| 101 | NM11 | Ethical approval for use of archival HMPS tissue was provided by the Southampton and South-West Hampshire Research Ethics Committee A (REC 06/Q1702/99). Ethical approval for the collection and use of endoscopic and archival TSA samples was obtained from the Oxfordshire Research Ethics Committee A (REC 10/H0604/72.) Informed consent for tissue use in research was obtained from all patients before endoscopic or surgical procedure. | Yes | - | Yes | - | - | Yes | - | - | - | Yes | - | Yes | - | - | Yes | - | - | - |
| 102 | NM12 | All human samples used in this study have been included in previous publications. All patients were recruited after providing informed consent and with approval by the participating institutional review boards, and the study was conducted according to the principles expressed in the Declaration of Helsinki. Written informed consent was obtaine from study participants or, for those with substantial cognitive impairment, from a caregiver, legal guardian or other proxy, and the study protocols for all populations were reviewed and approved by the appropriate institutional review boards. | Yes | - | Yes | - | - | Yes | - | - | - | Yes | - | - | Yes | - | Yes | - | - | - |
| 103 | NM13 | Human kidney tissue collection was approved by the University of Pennsylvania and Albert Einstein College of Medicine Institutional Review Board. | - | Yes | - | - | - | - | - | - | - | Yes | - | Yes | - | - | Yes | - | - | - |
| 104 | NM14 | Primary pediatric human glioma cells, designated “SF,” were obtained from surgical biopsy of tumor from patients admitted to UCSF Medical Center and in accord with an institutionally approved protocol by the UCSF Committee for Human Research (IRB# 10-01318). Subjects, or their legal guardians if the subject was a minor, provided informed consent. Cell line KNS42, with H3F3A G34V mutation (substitution of glycine 34 with valine)17, was obtained from the Japanese Collection of Bioresources and was established from a 16-year-old male. | Yes | - | Yes | - | - | Yes | - | - | - | Yes | - | Yes | - | - | Yes | - | - | - |
| 105 | NM15 | All study protocols have been approved by the ethics committee of the University of Leipzig. All participants gave written informed consent before taking part in the study. | Yes | - | - | Yes | - | Yes | - | - | - | Yes | - | Yes | - | - | Yes | - | - | - |
| 106 | NM16 | The study was approved by the Massachusetts General Hospital Institutional Review Board and Partners Healthcare Human Research Committee. A waiver of consent for discarded clinical material was obtained. | Yes | - | Yes | - | - | Yes | - | - | - | Yes | - | Yes | - | - | Yes | - | - | - |
| 107 | NM17 | The Boston Medical Center Institutional Review Board approved the study, and all participants provided written informed consent. | Yes | - | - | Yes | - | Yes | - | - | - | Yes | - | Yes | - | - | Yes | - | - | - |
| 108 | NM18 | Patients for gene expression analyses were selected among 591 individuals who received preoperative anthracycline-based, taxane-free chemotherapeutic regimens at the Gustave Roussy Cancer Campus between 1987 and 2003. The study was approved by the local Institutional Reviewing Board at the Institut Gustave Roussy (Villejuif, France). All patients signed an informed consent form for the storage and molecular analysis of their samples. In addition, 41 patients with newly diagnosed, biopsy-proven, stage II or IIIA ERBB2− BC were evaluated in a prospective study of neoadjuvant anthracycline-based chemotherapy at Georges François Leclerc Cancer Center (Dijon, France). This prospective study was approved by the Institutional Review Board at the Georges François Leclerc Cancer Center (Dijon, France), and all women gave their informed consent. We analyzed 497 tissue microarrays from the PACS04 phase 3 clinical trial (ClinicalTrials.gov ID NCT00054587), enrolling 3,010 patients with BC presenting with axillary lymph node involvement but no distant metastases between 2001 and 2004. Surgical specimens were available for 1,836 out of 3,010 participants in the study. These samples were collected centrally in the context of the UNICANCER initiative by the Department of Pathology of the Jean Perrin Cancer Center (Clermont-Ferrand, France) and were included in tumor TMAs. The PACS04 protocol was reviewed and approved by the ethics committee/institutional review board, and the study was conducted according to the Declaration of Helsinki and European Good Clinical Practice requirements. Patients signed an informed consent. | Yes | - | Yes | - | - | Yes | - | - | - | Yes | - | Yes | - | - | Yes | - | - | - |
| 109 | NM19 | All human specimens were processed in compliance with the institutional review board at the University of Pennsylvania and HIPAA requirements. | - | Yes | - | - | - | - | - | - | - | Yes | - | Yes | - | - | Yes | - | - | - |
| 110 | NM20 | All individuals donated samples following informed written consent under local ethics board–approved protocols 239/99_BG, 251/13_KW, and 282/11_SE version 140023 (Ethik Kommission der Albert-Ludwigs-Universität Freiburg) and protocols #04/Q0501/119_AM03 for affected individuals, #07/H0720/182 for family members and #08/H0720/46 for healthy controls (Royal Free Hospital & Medical School Research Ethics Committee, London). | Yes | - | - | Yes | - | Yes | - | - | - | Yes | - | Yes | - | - | Yes | - | - | - |
| 111 | NM21 | The study was approved by the Human Research Committee at Brigham and Women’s Hospital (Boston, MA), and participants provided written informed consent. | Yes | - | - | Yes | - | Yes | - | - | - | Yes | - | Yes | - | - | Yes | - | - | - |
| 112 | NM22 | Informed consent was obtained from all human subjects (or their parents). Protocols were approved by the East London and City Research Ethics Committee (cord blood samples, HREC: O6/Q0604/110); the South London Research Ethics Committee 2 (cohort of preterm babies, HREC: 10/H0802/40); the Wandsworth Research Ethics Committee (adult controls, HREC: 07/H0803/237); and the Bloomsbury Research Ethics Committee (NEC sections, HREC: 11/LO/0495). | Yes | - | - | Yes | - | Yes | - | - | - | Yes | - | Yes | - | - | Yes | - | - | - |
| 113 | NM23 | Units of human blood were obtained from Brigham and Women’s Specimen Bank (protocol number M20403-101). | - | Yes | - | - | - | - | - | - | - | - | Yes | - | - | - | - | - | - | - |
| 114 | NM24 | All patient tumor biopsy samples were obtained under Institutional Review Board (IRB)-approved protocols (Vanderbilt University IRB# 050644, Memorial Sloan Kettering Cancer Center IRB #10-136, University Hospital of Cologne IRB #06037 and Peter MacCallum Cancer Center IRB#08/71). Written informed consent was obtained from all patients. All samples were deidentified, and protected health information was reviewed according to Health Insurance Portability and Accountability Act (HIPAA) guidelines. | Yes | - | Yes | - | - | Yes | - | - | - | Yes | - | Yes | - | - | Yes | - | - | - |
| 115 | NM25 | For human AA samples, perilesional punch biopsies from 5 patients with patchy alopecia areata who were not undergoing local or systemic treatments were collected and compared to scalp biopsies from 5 unrelated unaffected individuals. All procedures were performed under Institutional Review Board–approved protocols at Columbia University and conducted under the Declaration of Helsinki principles. Informed consent was received before inclusion in the study. | Yes | - | Yes | - | - | Yes | - | - | - | Yes | - | Yes | - | - | Yes | - | - | - |
| 116 | NM26 | All subjects included in the study provided informed consent, and the project was approved by The Institutional Review Board of the Douglas Mental Health University Institute. | Yes | - | - | Yes | - | Yes | - | - | - | Yes | - | Yes | - | - | Yes | - | - | - |
| 117 | NM27 | From August 2012 to February 2013, 55 patients were recruited to our broader program of SCLC biomarker research. Patients had histologically or cytologically confirmed chemotherapy-naive SCLC and were referred to a tertiary cancer center, The Christie Hospital NHS Trust. The study was prospectively approved by the NHS NorthWest Research Ethical Committee. Clinical and demographic data were collected. During this period, we initiated our CDX study, and 11 patients provided additional informed consent that specified their samples could be used for in vivo studies and genetic analysis in accordance with UK regulatory requirements. | Yes | - | Yes | - | Yes | - | Yes | - | - | Yes | - | Yes | - | Yes | - | - | - | Yes |
| 118 | NM28 | This study was approved by the Institutional Review Board Committee at the Baylor College of Medicine (Houston, TX, USA). Written informed consent was obtained from the parents of our subjects. Primary cultures of genital skin fibroblasts (GSF) isolated from the foreskin of male neonates at the time of circumcision or surgical correction were obtained from patients with hypospadias and/or cryptorchidism after approval from the Baylor College of Medicine Institutional Review Board (Houston, TX, USA). | Yes | - | - | Yes | - | Yes | - | - | - | Yes | - | Yes | - | - | Yes | - | - | - |
| 119 | NM29 | All patients provided written informed consent, and the approval for the study was provided by the Institutional Review Board of the University of Texas M.D. Anderson Cancer Center, the Institutional Review Board of the University of California, San Diego, and the ethics committees of the Changhai Hospital, the Second Military Medical University, the Fudan University–affiliated Zhonghan Hospital and the Tongji Hospital, Shanghai, China. | Yes | - | - | Yes | - | Yes | - | - | - | Yes | - | Yes | - | - | Yes | - | - | - |
| 120 | NM30 | Plasma samples were obtained from antiretroviral-naive mother-infant pairs enrolled in the Nairobi Breastfeeding Trial11. The ethical review committee of the Kenyatta National Hospital Institutional Review Board, the Institutional Review Board of University of Washington and the Institutional Review Board of the Fred Hutchinson Cancer Research Center approved the study. In addition, the Kenyan Ministry of Health gave permission to conduct the Nairobi Breastfeeding Trial. Informed consent was obtained from all participants. | Yes | - | - | Yes | - | Yes | - | - | - | Yes | - | Yes | - | - | Yes | - | - | - |
