# Supplementary material for: Ethics Reporting in Biospecimen and Genetic Research: Current Practice and Suggestions for Changes
Source: PLoS Biol. 2016 Aug 2;14(8):e1002521. doi: 10.1371/journal.pbio.1002521 (PMC4970810; doi:10.1371/journal.pbio.1002521)
Supplement: S2 Table — (DOCX) [file pbio.1002521.s002.docx]

The following examples were rated as non-meaningful ethics reporting because they do not describe whether the consent was broad or study-specific, nor whether the approval was for banking and/or a single project that disposed of remaining samples at the end of the study. Without further information, one can only speculate about the content and context of the consent and approval.

| Examples |
| --- |
| “All cases and controls were enrolled in the study following informed consent and ethical approval from the relevant national and regional institutional review boards for each sample collection”. (NG18, S1 Table) |
| “Patients, parents, or guardians gave written informed consent for sample collection and research, with assent provided by older children and adolescents. The study was approved by the St. Jude Institutional Review Board”. (NE22, S1 Table) |
| “All patients signed an informed consent form for the storage and molecular analysis of their samples”. (NM18, S1 Table) |
| “Crohn’s disease patients for the sequencing experiment (n = 474) were recruited from specialist IBD clinics in London and Newcastle [37] after informed consent and ethical review (REC 05/Q0502/127). Population controls for sequencing (n = 480) were obtained from the 1958 British Birth Cohort [38]” (PG4, S1 Table) |
