# Supplementary material for: Ethics Reporting in Biospecimen and Genetic Research: Current Practice and Suggestions for Changes
Source: PLoS Biol. 2016 Aug 2;14(8):e1002521. doi: 10.1371/journal.pbio.1002521 (PMC4970810; doi:10.1371/journal.pbio.1002521)
Supplement: S1 Text — (DOCX) [file pbio.1002521.s003.docx]

We guessed that we might find the most detailed and meaningful ethics reporting in leading journals for genetics and medicine that have strict editorial policies on ethics reporting in general. We therefore chose the following four journals: PLoS Genetics, New England Journal of Medicine, Nature Genetics, and Nature Medicine. For each journal, we performed a literature search on PubMed using the following search expression: ("title abbreviation"[Journal] AND ("2014/06/01"[PDat]: "2015/06/30"[PDat]) AND Humans[Mesh] NOT "review"[publication type]). Since this search yielded 107 articles for PLoS Genetics, 170 for Nature Genetics, and 191 for Nature Medicine, but 1381 for New England Journal of Medicine, we further narrowed the search in the last journal by excluding additional article types, such as comment, editorial, letter and case reports, which resulted in 239 articles. For all retrieved references, titles and abstracts were screened for relevance. For each journal the first 30 relevant articles were included. Studies were judged relevant if they reported findings from any scientific analysis of human biospecimens. Biospecimens were defined as any human material comprised of whole solid tissues, cells isolated from solid tissues, and fluids, including blood. From the resulting 120 full text articles, all text passages relevant to ethics reporting were extracted. Two researchers with training and practical experience in basic science research (WC, SW) extracted relevant information from 60 articles each. All five authors developed and agreed a matrix assessing each paper in the two categories “consent” and “approval”. For each category, we distinguished further whether: A) there was any reporting at all, B) any details were reported and C) these details included sufficient information on the type of consent obtained from donors (broad, project-specific or both), and of approval (for banking of biospecimens, project-specific use, or both).

Based on this matrix and the extracted information, two researchers (WC, SW) rated 60 articles each, one researcher (DS) rated all 120 articles, and two researchers (JP, TI) rated a randomized sample of 20 articles each (5 from each journal). All initial disagreements between authors were resolved by discussion. The resulting data were then descriptively analyzed and summarized.

A limitation of our study was that the ratings of the ethics statements included interpretive judgments which could affect the validity and reliability of the results. We addressed this by having at least two researchers with expertise in basic research and research ethics rate each article and two other researchers with expertise in biobank research confirm the rating evaluation of a randomized sample. Furthermore, we rated an ethics statement as providing meaningful information on consent or approval where that information could be inferred, even if it did not address consent or approval explicitly. Readers can assess our study validity, as SI1 presents all 120 ethics reports and our ratings.
